# Human Adipose-Derived Stem Cell Conditioned Media and Exosomes Containing *MALAT1* Promote Human Dermal Fibroblast Migration and Ischemic Wound Healing

**DOI:** 10.1089/wound.2017.0775

**Published:** 2018-09-04

**Authors:** Denise R. Cooper, Chunyan Wang, Rehka Patel, Andrea Trujillo, Niketa A. Patel, Jamie Prather, Lisa J. Gould, Mack H. Wu

**Affiliations:** ^1^ Research Service, James A. Haley Veterans Hospital, Tampa, Florida.; ^2^ Department of Molecular Medicine, University of South Florida Morsani College of Medicine, Tampa, Florida.; ^3^ Department of Physiology and Pharmacology, University of South Florida Morsani College of Medicine, Tampa, Florida.; ^4^ Department of Surgery, University of South Florida Morsani College of Medicine, Tampa, Florida.

**Keywords:** lncRNA, *MALAT1*, exosomes, wound closure

## Abstract

**Objective:** Chronically ill patients heal recalcitrant ulcerative wounds more slowly. Human adipose-derived stem cells (hADSCs) play an important role in tissue regeneration and exosomes secreted by hADSC contribute to their paracrine signaling. In addition to cytokines, lipids and growth factors, hADSC secrete mRNA, miRNA, and long noncoding (lnc) RNA into exosomes. In this study we examined the role of lncRNA *MALAT1* (metastasis-associated lung adenocarcinoma transcript 1), an abundant lncRNA in exosomes from conditioned media (CM), on cell migration and ischemic wound healing.

**Approach:** CM and isolated exosomes from hADSC were applied to human dermal fibroblast (HDF) in scratch assays and electric cell-substrate impedance sensing (ECIS) assays. CM was also applied to a rat model of ischemic wound healing and wound closure was followed.

**Results:** CM stimulated cell migration of HDFs *in vitro* by 48%. CM stimulated the closure of ischemic wounds in a rat model 50% faster than unconditioned media. The depletion of *MALAT1* in adipose-derived stem cell (ADSC) CM significantly reduced cell migration. Since *MALAT1* is secreted into exosomes, a purified population of exosomes was applied to HDF where they enhanced cell migration in a similar manner to FGF-2 or basic fibroblast growth factor (bFGF) in ECIS wound healing assays. The uptake of exosomes by HDF was shown using dynasore, an inhibitor that blocks clathrin- and caveolin-dependent endocytosis. Depletion of *MALAT1* in hADSC with antisense oligonucleotides resulted in exosomes without *MALAT1*. These exosomes had an effect similar to the unconditioned, control media in ECIS assays.

**Innovation:** Exosomes contain lncRNA *MALAT1* and other factors that have the potential to stimulate HDF cell migration and angiogenesis involved in wound healing without applying stem cells to wounds.

**Conclusion:** Our results show the potential of using topically applied ADSC-derived exosomes containing *MALAT1* for treating ischemic wounds. This allows for harnessing the power of stem cell paracrine signaling capabilities without applying the cells.

**Figure:**
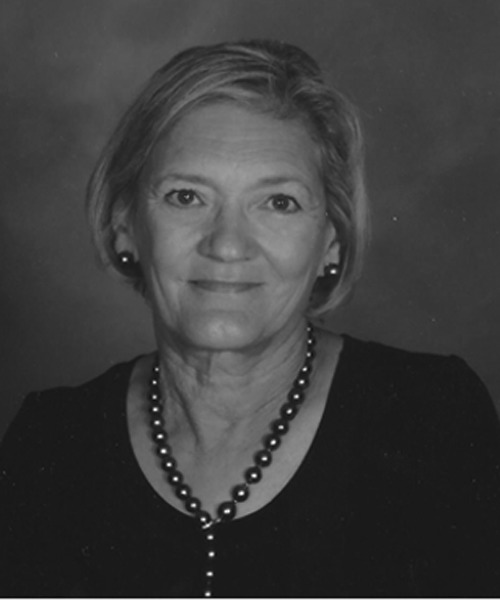
**Denise R. Cooper, PhD**

## Introduction

Chronically ill patients are faced with challenges when they develop recalcitrant wounds. A number of local and systemic factors impact wound healing including oxygenation, infection, age, sex hormones, stress, diabetes, obesity, medications such as chemotherapy, alcoholism, smoking, and nutrition.^1^ The wound healing process consists of four integrated and overlapping phases: hemostasis, inflammation, proliferation, and tissue remodeling.^2^ These must occur rapidly and appropriately in the proper sequence and continue for a specific duration for wound healing to occur successfully.^3^ Investigations and clinical studies have provided a wealth of information about normal and impaired wound healing. Proper oxygen levels are crucial for optimum wound healing. Hypoxia (low oxygen) stimulates the release of growth factors and angiogenesis, while oxygen is needed to sustain the process.^4^ Inflammation, a normal part of wound healing is important in the removal of micro-organisms. Incomplete removal of bacteria and endotoxins can lead to infections at the injured skin surface, lengthening the inflammatory phase. Prolongation and escalation of the inflammatory phase contributes to a failure to heal. It also leads to increased levels of matrix metalloproteinases that can degrade the extracellular matrix. Systemic factors also contribute to impaired wound healing.^1^

The needs of a healing wound are complex. Various factors play a role in mechanisms that contribute to the overall outcome. Mesenchymal stem cells (MSCs) derived from bone marrow improve wound fibroblast migration *in vitro.*^5^ However, an *in vivo* model of injury, preferably one of impaired wound healing, is crucial for demonstrating the healing properties of any treatment.^6,7^ Thus, an ischemic wound model using adult rats was implemented to assess the efficacy of conditioned media (CM) from adipose-derived stem cells (ADSCs) on the closure of ischemic wounds versus nonischemic wounds in the same animal. In addition, the scratch assay, a straightforward method to measure the rate of cell migration *in vitro* using human dermal fibroblasts (HDFs), evaluated CM *in vitro*. Earlier studies cocultured MSCs with fibroblasts to determine a role for soluble factors and cell to cell interactions and showed in Boyden chambers that fibroblasts migrated toward MSCs in the chamber.^5^ We used the ADSC-derived CM to demonstrate that stem cell secreted factors stimulated fibroblast migration. We followed this up with an animal model to assess wound closure, and focused on a specific long noncoding (lnc) RNA, *MALAT1* (metastasis-associated lung adenocarcinoma transcript 1), which modulated healing in a model of traumatic brain injury (TBI).^8^ Exosomes, specific nanovesicles secreted by stem cells, were isolated to demonstrate the ability of secreted stem cell factors in exosomes to increase cell migration. Exosomes are of interest due to their cargo proteins, lipids, mRNA, lncRNA, and miRNA. Exosomes evade immune rejection by the host and modify cellular responses.^9^ We demonstrate that ADSC-derived exosomes increase wound healing by stimulating the migration of dermal fibroblasts, and that the lncRNA, *MALAT1*, is largely responsible for increased cell migration *in vitro*.

## Clinical Problem Addressed

This study focuses on exosomes, extracellular vesicles secreted by stem cells, which, when isolated from CM, can alter the response of wounded cells, and increase *in vivo* wound healing. The contents of exosomes include lncRNA *MALAT1* that increases cell migration to mediate healing of ischemic wounds. The uptake of exosomes by wounded cells is required. By treating human adipose-derived stem cell (hADSC) with antisense oligonucleotides (ASO) to *MALAT1*, exosomes secreted by ADSC are depleted of *MALAT1*, and this preparation of exosomes failed to stimulate cell migration. Exosomes provide a therapeutic model for addressing the process of wound healing in a number of cellular settings.

## Materials and Methods

### Adult HDF culture

Adult-HDF were purchased from ScienCell (Catalog No. 2320). HDF, isolated from human skin, were negative for HIV-1, HCV, mycoplasma, bacteria, yeast, and fungi. Cells were expanded in fibroblast medium (FM, Cat No. 2301) according to manufacturer's protocol. Cells were subcultured in poly-L-lysine-coated culture vessels as recommended. Medium was changed every 3 days after plating until cells were 70% confluent and every other day until cells were 90% confluent.

### ADSC culture and CM

Human ADSC were purchased from ZenBio, Inc. Cells were negative for HIV-1, HIV-2, HTLC-1, HTLV-2, Hep-B, Hep-C, and mycoplasma. Human ADSC were isolated from subcutaneous adipose tissue of normal, nondiabetic donors between 25 and 45 years of age undergoing elective surgery. They were cultured according to manufacturer. For CM collection, cells were grown to 90% confluence, media were replaced with chemically defined serum-free mesenchymal stem cell basal media (MSCBM) (Lonza™), and CM was collected after 48 h.

### Exosome isolation

Exosomes were isolated using a two-step protocol. First, ExoQuick™ solution (Systems Biosciences) was added to culture media at a volume of one to five. Following centrifugation at 1,500 *g* for 30 min, the pellet was further processed. ExoCap™ (JSR Life Sciences) composite reagent containing magnetic beads for CD9, CD63, and CD81 was used to purify exosomes. Exosomes were eluted from beads using modified manufacturer's elution buffer (M) and used in wound healing assays after washing twice with 500 μL washing buffer.^10^ To deplete *MALAT1* from exosomes, ADSC were transfected with ASO as described.^11^ Scrambled ASO were transfected as a control into ADSC, and CM was collected as described above. Exosomes were quantitated using a NanoSight LM10 (Malvern Instruments), and showed an average size of 90–100 nm at a concentration of 1.1 × 10^8^ per mL from 10^6^ cells CM.^10,11^

### Scratch assays

HDF were plated in 24-well plates. The cells were grown to confluence and 3 days after confluence were mechanically disrupted with a sterile 200 μL pipette tip with the use of a grid of 3 × 3 mm squares scratched with a pipette tip as described.^8^ The preincubation with mitomycin C (10 μg/mL) for 2 h blocks further proliferation so that only migration is followed.

### Electric cell-substrate impedance sensing assays

The electric cell-substrate impedance sensing (ECIS) instrument (Applied Biophysics) was used to measure the impedance on electrically wounded hHDF to detect the migratory response. Briefly, 200 μL of cell suspension was seeded in each well of 8W1E ECIS array (5 × 10^4^ cells per well) coated with 1% gelatin. After cells reached confluency in the incubator, the ECIS chambers were mounted to the ECIS system. Once the baseline leveled off, a 240 mA current with 60 kHz frequency was applied to the cell-covered electrode for 30 s to kill the cells on the electrode (250 μm diameter), which resulted in impedance decreasing to around 2,000 ohms. Arrays were then washed with medium under the microscope to remove any dead cells on the electrodes. Afterward, HDF were treated with mitomycin C as described above. Exosomes were added to wells, and the wound healing process was determined in real-time by measuring the recovery of transcellular electrical resistance (TER) impedance, an indicator of the surrounding viable fibroblast cells migrating into the wounded area, which was measured in ohms.

### Rat model of ischemic wound healing

Animal procedures were approved by the Animal Care and Use Committee at the University of South Florida and abided by all requirements of the Animal Welfare Act and the Guide for Care and Use of Laboratory Animals. Six-month-old male Fischer 344 rats (National Institute on Aging, Bethesda, MD) were utilized to create an ischemic wound model.^6^ Full-thickness excisional wounds were created in the center of a 10.5 × 3.5-cm flap (ischemic wounds) with a 6 mm punch biopsy and wound healing were measured at day 2, 5, 7, 10, 14, and 21. Control (nonischemic) full-thickness wounds were created on either side of the ischemic flap for comparison. At the time of wounding and upon harvest, six rats were anesthetized, ischemic and nonischemic wounds were digitally photographed, and wound sizes were determined.^7^ CM from ZenBio™ ADSC (20 μL) or control (unconditioned) media (20 μL) was applied to each wound daily. Wound sizes were determined on day 0, 2, 5, 7, 10, and 21.

### Statistical analysis

PRISM-6™ software was used for statistical analysis with appropriate tests for comparisons including one-way ANOVA and unpaired *t*-test. Data are shown as mean ± SEM. An *n* = 3 is shown unless stated otherwise.

## Results

### CM from ADSC promoted HDF cell migration

**Figure 1.**
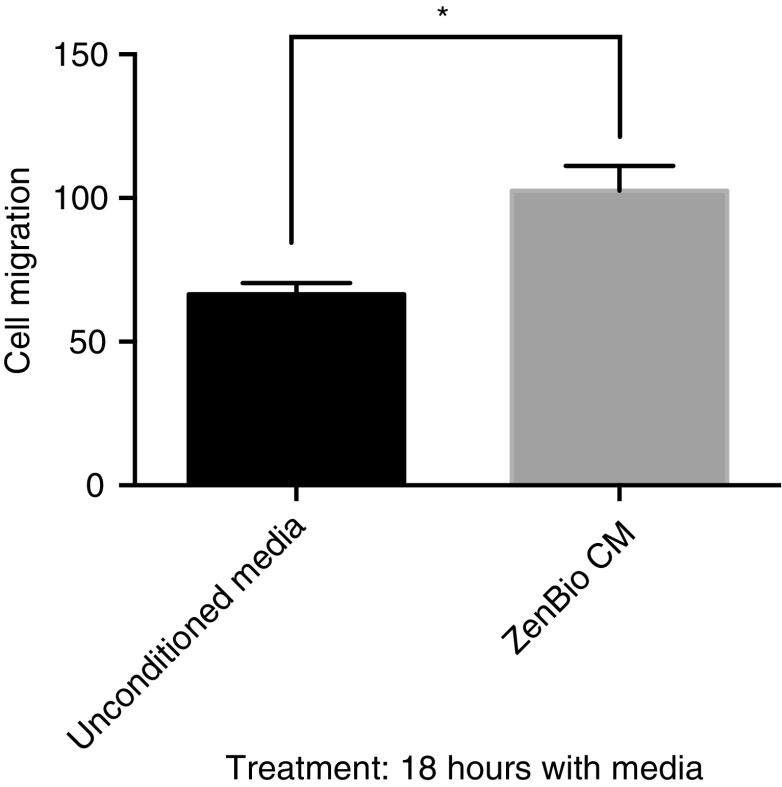
CM from ADSC promoted cell migration in HDF. HDFs were plated in six-well dishes and when 95% confluent, cells were scratched (3 × ) with a 200 μL pipette. Cells were treated with mitomycin C for 2 h. Cell media from ZenBio™ ADSC were added (2 mL) for 18 h. Unconditioned media (Lonza MSCMCD) were added as the control. After capturing images, migrating cells were counted in a field of the three scratch areas. PRISM™ analysis of data indicated *p < 0.05. ADSC, adipose-derived stem cell; CM, conditioned media; HDF, human dermal fibroblast.

Fibroblasts interact with their environment to produce a migratory mode in wound healing. There are complex networks that occur through signals that drive the processes in wound healing. The *in vitro* scratch assay is a convenient method for analysis of cell migration to assess the effects of exogenous factors on migration of individual cells.^12^ To probe for secreted factors from ADSC that modulate cell migration, we collected CM from ZenBio subcutaneous derived ADSC. CM was collected under stringent conditions using a serum-free, clinical grade media. This 48 h collection was termed ZenBio CM. ZenBio CM was compared to control, or unconditioned Lonza MSCBM. CM increased the migration of HDF in the scratch assay by 43% (Fig. 1). This result indicated that ZenBio CM contained factors that increased cell migration.

### CM from ADSC closed ischemic wounds in Fischer 344 rats

**Figure 2.**
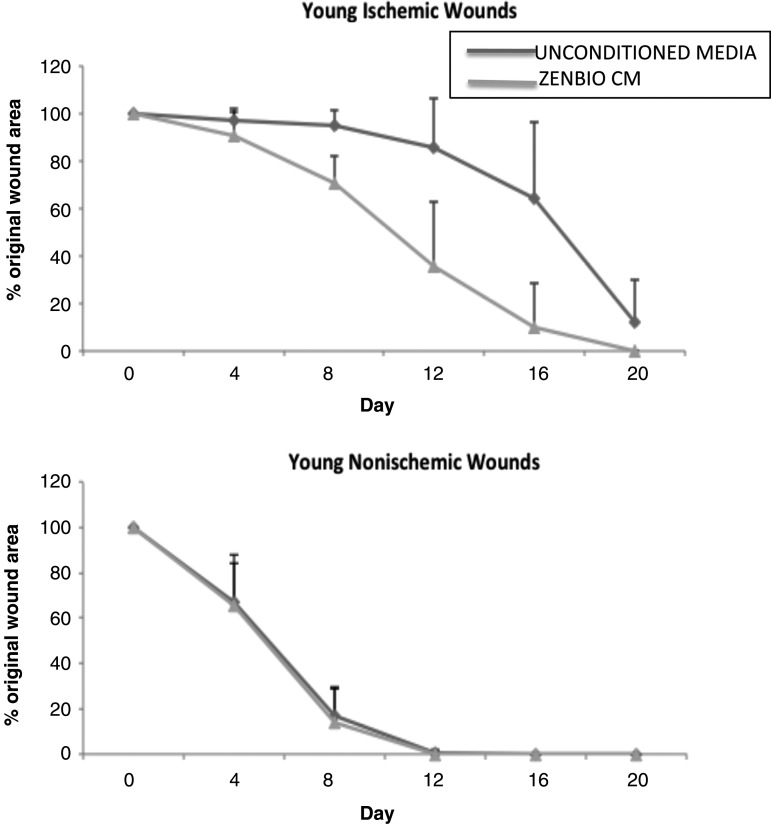
CM from ADSC closed ischemic wounds in Fischer 344 rats. Twenty-four Fisher rats underwent creation of a bipedicled ischemic flap with 6-mm excisional wounds, two ischemic and two nonischemic (control wound). Adult (6 month old) rats were divided into groups of 6, comparing human ADSC-CM obtained from normal donors (ZenBio). Twenty microliters of CM was applied topically daily to each wound beginning on the day of surgery. Digital photographs were taken every 4 days and wound area measured using Image J. Analysis revealed a statistically significant difference between control and lean CM in ischemic wound sizes for days 8, 12, and 16 (*p* < 0.001). One hundred percent of lean CM treated ischemic wounds were healed at day 20, 50% of control media.

The *in vitro* results using CM from ADSC suggested that fibroblast migration, which plays a key role in wound closure was accelerated. Next, we applied ZenBio hADSC CM to rat ischemic wounds to determine whether our *in vitro* observation would translate to an *in vivo* model. We applied ZenBio CM to ischemic and nonischemic wounds for 20 days and measured the percent of wound closure that occurred in each wound (Fig. 2). Our results demonstrated that in adult rats, ZenBio CM accelerated closure of ischemic wounds. By day 4, there was a 66% wound area reduction of nonischemic (control) wounds. By day 8, this increased to 86% wound area reduction, and by day 12, nonischemic wounds were healed. There was no difference between unconditioned media or ZenBio CM for non-ischemic wounds. For ischemic wounds, ZenBio CM treated wounds were closed 10% on day 4, 30% on day 8, and by day 12, the wound area reduction was 65% compared to only 15% wound closure for the control (unconditioned) media treated wounds. Thus, ZenBio CM increased ischemic wound closure by 50% compared to nonischemic wounds. This is more robust than the results of the scratch assay reported above.

### Exosomes from CM stimulated cell migration in ECIS assays

**Figure 3.**
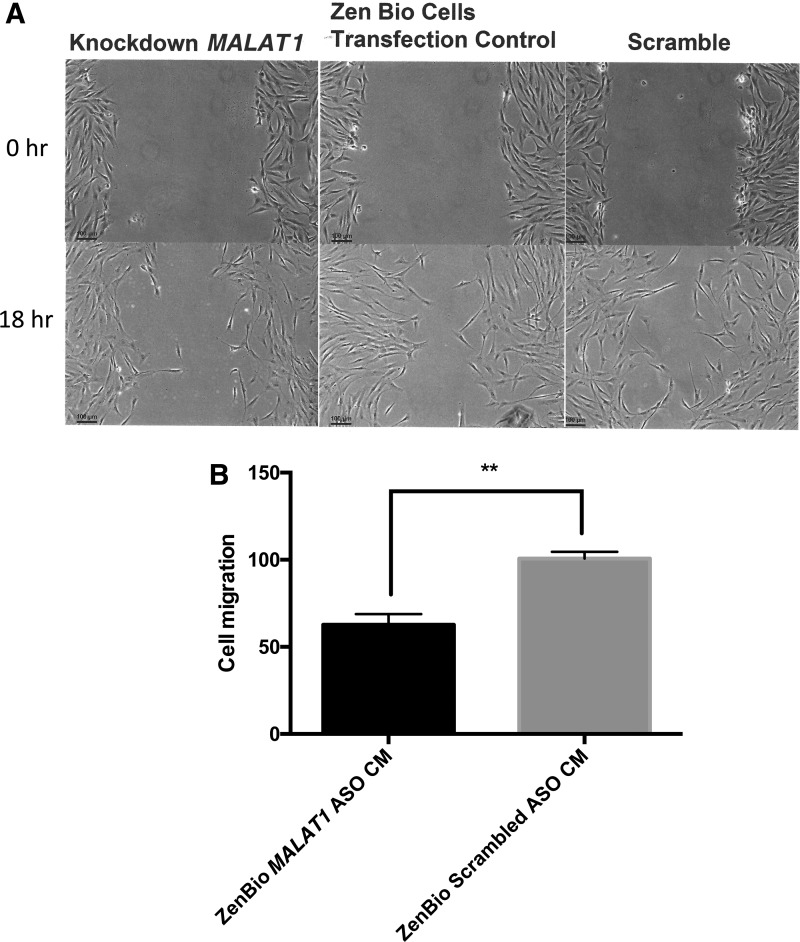
*MALAT1* depletion in CM blocked cell migration. HDFs were plated in six-well dishes and when 95% confluent, cells were scratched (3 × ) with a 200 μL pipette. Cells were treated with mitomycin C for 2 h. Cell media from *MALAT1* ASO treated or “scrambled” ASO treated ADSC were added (2 mL) for 18 h. **(A)** After capturing images, migrating cells were counted in a field of the three scratch areas. **(B)** PRISM analysis indicated **p < 0.01. ASO, antisense oligonucleotides; *MALAT1*, metastasis-associated lung adenocarcinoma transcript 1.

We previously showed that *MALAT1* depletion of ZenBio CM suppressed the recovery of rats injured with TBI.^13^ In these studies, CM was injected into the rats after injury and their ability to swim a water maze improved, and ZenBio CM depleted of *MALAT1* impaired the improvement. Here, the effect of depletion/reduction in CM *MALAT1* was tested in HDF. Depletion of *MALAT1* in exosomes was accomplished using ASO to treat ZenBio hADSC with subsequent collection of CM. HDF treated with *MALAT1*-depleted CM resulted in reduction of cell migration of 48% compared to CM from hADSC treated with the scrambled ASO (Fig. 3).

CM contains numerous ADSC secreted factors. The secretome of ADSC from CM contains a number of highly conserved proteins that have roles in angiogenesis, regeneration, and extracellular matrix remodeling.^14^ In previous studies, exosomes derived from hADSC were found to accelerate cutaneous wound healing.^15^ In that study exosomes were injected intravenously and applied topically. The effect was thought to be via their RNAs and proteins. We found that exosomes isolated from CM contained large concentrations of *MALAT1* lncRNA.^10,11^ *MALAT1* was shown to increase cell migration in lung cancer.^16^ Whether it could increase cell migration of wounded HDF was tested next.

### Exosomes enhance cell migration

**Figure 4.**
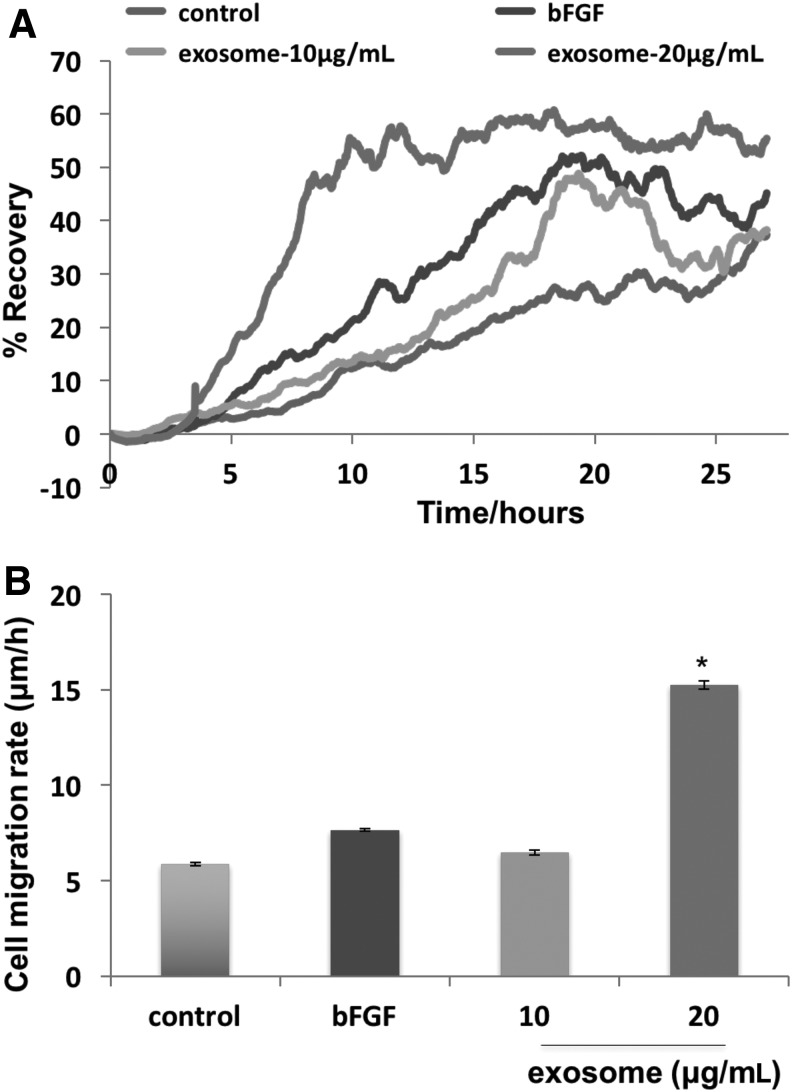
Exosomes from hADSC CM promoted HDFa cell migration in ECIS wound healing assay. HDFa were grown to confluency on eight well ECIS plate and cells were killed using electrical voltage as described in the Materials and Methods section. Cells were then treated with control (unconditioned) media, bFGF (400 nM), 10 μg/mL exosome, and 20 μg/mL exosome. **(A)** Resistance values were measured every 120 s and% recovery was calculated as the difference in the resistance value of each time point and the resistance value of the first time point after wounding, divided by the resistance before wounding. Treatment with 10 μg/mL exosome increased recovery of TER mimicking the effect of cells treated with bFGF. Treatment with 20 μg/mL exosome further enhanced the recovery of TER compared to cells treated with bFGF. *n* = 3. **(B)** Cell migration rate was determined by formula *v* = *r/t* where *r* = 125 micrometer (radius of the microelectrode) and *t* = time to heal. *n* = 3. *p < 0.05 from control. Wounded HDFa cells treated with 20 μg/mL exosomes showed a significant twofold increase in cell migration compared to the bFGF treatment. bFGF, FGF2 or basic fibroblast growth factor; ECIS, electric cell-substrate impedance sensing; hADSC, human adipose-derived stem cell; TER, transcellular electrical resistance.

To gain more insight than the semi-quantitative scratch assay provided, we used ECIS wound assay to measure recovery of TER and cell migration rate after wounding in cultured HDF. The concentration of exosomes applied was roughly equal to the number of exosomes found in CM. For this assay, we used FGF-2 or basic fibroblast growth factor (bFGF) (400 nM) as a positive control since it is known to promote cell migration via the ERK1/2 and JNK pathways.^17^ We then administered control, unconditioned media and two doses of exosomes suspended in control media to injured cells. Figure 4A demonstrates that the lower dose of exosomes increased recovery of TER similar to the effect of cells treated with bFGF. The higher concentration of exosomes enhanced the recovery of TER faster and higher than the effect of bFGF and promoted cell migration rate in a similar fashion (Fig. 4B. This indicated that exosomes promoted a strong effect on cell migration in ECIS wound healing assays.

### Dynasore blocked the effect of exosomes

**Figure 5.**
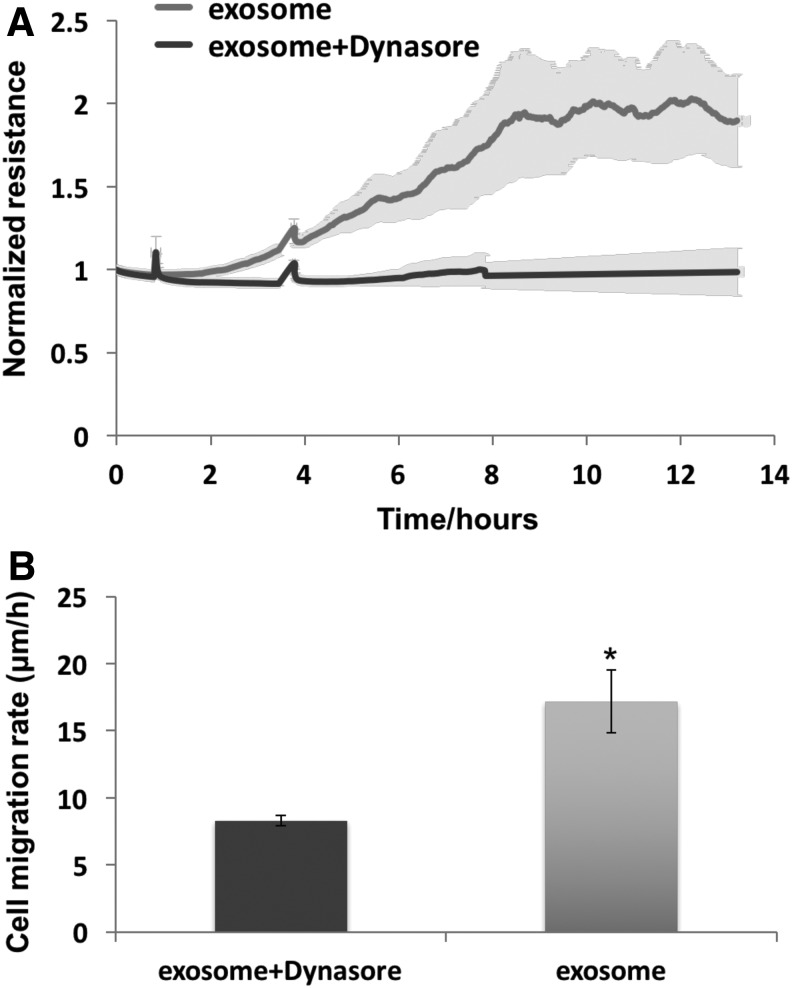
Dynasore blocked the effect of exosomes on HDFa cell migration in ECIS wound healing assay. HDFa were grown to confluency on eight well ECIS plate and then killed with a high electrical voltage as described in Materials and Methods section. Subsequently, the cells were treated with 20 μg/mL exosomes with or without dynasore (50 μM) respectively. **(A)** Resistance was measured every 120 s. The increase of TER in the cells treated with dynasore was significantly greatly attenuated compared to the cells without dynasore. *n* = 3. **(B)** Dynasore significantly decreased the rate of cell migration to the wounded microelectrodes as determined using formula *v = r/t* (*r* = 125 micrometer, radius of microelectrode and *t* = healing time). *n* = 3 and *p<0.05.

Extracellular exosomes have shown evidence that they can enter cells and deliver their cargo.^15^ The inhibitor, dynasore, was added to wounded cells to determine whether it blocked a clathrin- and caveolin-dependent endocytosis of exosomes by the HDF. Dynasore (50 μM) addition to the exosome-containing media greatly attenuated the increases of TER (Fig. 5A) and cell migration rate (Fig. 5B) in response to exosomes.

### Exosomes isolated from *MALAT1*-depleted CM failed to enhance cell migration

**Figure 6.**
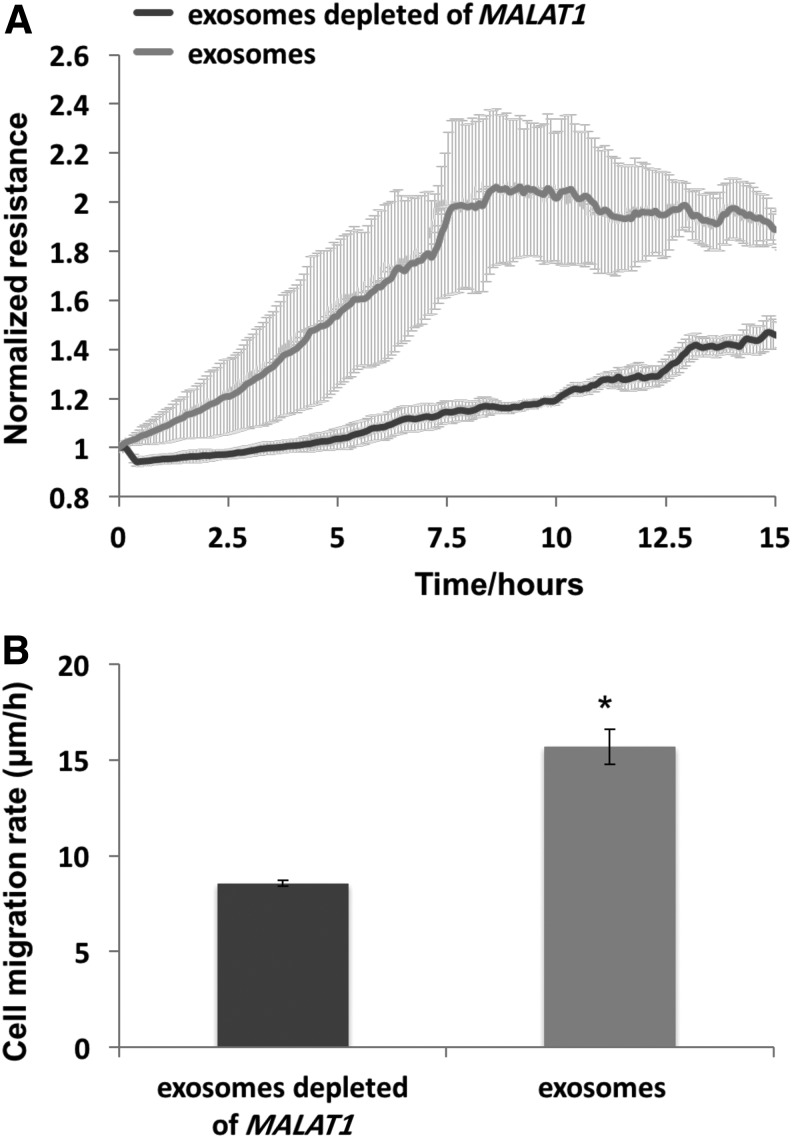
Exosomes from *MALAT1* depleted hADSC CM failed to enhance HDFa cell migration in ECIS wound healing assay. HDFa were grown to confluency on eight-well ECIS plate before killing using a high electrical voltage as described in Materials and Methods section. Cells were then treated with exosomes isolated from hADSC CM and exosomes isolated from *MALAT1* depleted hADSC CM, respectively. **(A)** Resistance values were measured every 120 s and the values were normalized to time 0 after wound. The increase of TER in the group treated with exosomes from hADSC CM depleted of *MALAT1* was significantly reduced compared to the group without *MALAT1* depletion. **(B)** Exosome media depleted of *MALAT1* significantly decreased the rate of cell migration as determined using formula *v = r/t* (*r* = 125 micrometer, radius of microelectrode and *t* = healing time). *n* = 3 and *p<0.05.

The loss of function of *MALAT1* in scratch assays was further demonstrated using exosomes derived from CM of hADSC where *MALAT1* was depleted with ASO. The results of ECIS wound assay displayed marked attenuation for recovery of TER in the cells treated with CM depleted *MALAT1* compared to complete CM over a 16-h course (Fig. 6A). The cell migration rate treated with CM without *MALAT1* was reduced 50% (Fig. 6B). The result demonstrated the importance of coexisting exosomes with *MALAT1* in promoting cell migration and wound enclosure.

## Discussion

The *in vivo* wound healing process is dynamic and consists of four continuous phases. In humans the release of proinflammatory cytokines such as bFGF is documented to occur before proliferation of fibroblasts within the wound bed to support angiogenesis.^18^ We tested treatment with ZenBio CM to determine whether ischemic wounds heal at a greater rate than those treated with unconditioned or “control” media. Previously, skin fibroblast migration was shown to be increased by the application of bone marrow-derived MSC,^19^ and adipose-derived MSC.^20^ These procedures require the isolation and engraftment of stem cells into the wound bed to be optimal.^21–23^ We noted that CM increased the rate of healing of HDF by a significant 48%. This effect was mimicked in an animal model of ischemic wounds with results at days 12 and 16 that mirrored the effect of CM on cells (50% closure). Since our previous studies in rats suffering from TBI indicated that the lncRNA *MALAT1* increased recovery of animals in the water maze,^13^ we depleted the levels of *MALAT1* in hADSC using ASO from Ionis Pharmaceuticals. These hADSCs produced CM that demonstrated a >70–90% reduction in *MALAT1* content as shown previously.^11^ We tested this CM in scratch assays and found that CM minus *MALAT1* did not promote cell migration and the control media or CM from scrambled-ASO treated hADSCs.

ECIS assays monitored real-time recovery of cell confluence after electrically creating a wound by measuring the resistance of the wound area as resistance (ohms) versus time measured in these assays. ECIS assays demonstrated the ability of ADSC-derived exosomes, isolated from CM, to increase migration of wounded cells. Migration was blocked using exosomes from CM of ADSC treated with *MALAT1* ASO.

Previously, we showed that exosomes contain substantial concentrations of *MALAT1* when isolated from ADSC-derived CM.^10,11^ The diminished expression of *MALAT1* in stem cells treated with ASOs targeting this lncRNA resulted in a reduced secretion of *MALAT1* into exosomes that was less than 80% of control exosomes. *MALAT1* depleted exosomes performed equally to “control” media in ECIS assays. This mirrored the effect of *MALAT1* ASO treated ADSC CM in scratch assays. Hence, *MALAT1* provided a critical function in HDF migration in both assays evaluating CM and exosomes. Moreover, *MALAT1* was taken up by the HDF as demonstrated by inhibition of migration when cells were treated with dynasore, a blocker of clathrin- and caveolin-dependent endocytosis where dynamin-2 is the target.^24,25^ The precise molecular mechanisms of *MALAT1* action in exosomes have yet to be determined. Its nuclear role is in alternative splicing of pre-mRNA.^11^ The effect of exosomes released from human induced pluripotent stem cell (iPSC) from MSCs was shown to promote collagen synthesis and angiogenesis *in vitro* and *in vivo.*^26^ Exosomes promoted proliferation and migration of human umbilical vein endothelial cells. This occurred in a dose-dependent manner where 100 μg/mL of exosomes was used. Here, we used 10-fold lower concentrations of exosomes to promote migration. This could be due to the specific cargo of the ADSC exosomes versus iPSC exosomes. We focused on lncRNAs and found that *MALAT1* in exosomes was extremely abundant.^10^ The lncRNA content of human iPSC from MSCss was not reported. Although HELA and MCF7 cells secrete *MALAT1*, these are cancer/tumor cells. *MALAT1* activity in cell migration is shown in these cells and *MALAT1* levels are upregulated.^27^

LncRNAs like *MALAT1* show low expression levels in cells, but they are enriched in exosomes of cancer cells.^28^ Much of the information on *MALAT1*'s function is revealed by cancer cell growth. In most models, *MALAT1* acts as a sponge for miRNAs, and include MiR-200c in endometrioid endometrial carcinoma (EEC),^29^ where *MALAT1* influenced EEC migration. In a uveal melanoma cell line, *MALAT1* promoted cell migration through modulating miR-140 expression.^30^ In another study, *MALAT1* upregulated the expression of miR-22-3p and its target genes CXCR2 and Akt.^31^ This resulted in protecting cells from ox-LDL-induced endothelial dysfunction by acting as a sponge. Hence, the role of *MALAT1* to act as a miRNA sponge is a recurring mechanism of its action in cell migration and probably applies to wound healing. Silencing of *MALAT1* can also occur by miR-101 and miR-217 to inhibit cell migration in esophageal squamous cell carcinoma cells.^32^ Another mode of action could be via transcription factor regulation. RUNX1 depletion led to upregulation of genes associated with chromatin structure and downregulation of genes related to extracellular matrix biology, and lncRNA *MALAT1*.^33^ The fine-tuning of local interactions is suggested, but it appears that exosomal lncRNAs have varied functions now emerging including signaling and acting as decoys or sponges.

Of interest is the origin of *MALAT1* in the secretome. Here, the source is a MSC residing in adipose tissue. It is not derived from cancerous cells, which may or may not secrete large amounts of *MALAT1* into vesicles. It is notable that MSC have antitumor activity when they are engulfed by cancer cells.^34^ The MSC secretome is known to be regenerative and modulate cancer cell activity.^35^ The secretome of MSC isolated from various tissues may diverge, but it is known to include cytokines such as TGFb, VEGF, IL-6, and more.^35^ This suggests that the total cargo of exosomes likely increases cell migration in a concerted manner. However, the importance of *MALAT1* is high as it accounts for much of the cell migration reported here.

Overall, increased *MALAT1* expression is deleterious to cancer metastasis due to cell migration, but its application to wounds via exosomes derived from stem cells appears therapeutic. This function of *MALAT1* in wound healing may prove to be the upregulation of collagenase, angiogenesis, and migratory genes.^36–38^ It would likely entail the interaction with different miRNAs to accomplish this. In all, there are hundreds of miRNAs that can interact with *MALAT1*. Interactions with *MALAT1* in the exosome would be the most important to elucidate, since they have been transported out of the stem cells to maintain stem cell function. The manifestation of enhanced wound healing due to the noncanonical functions of *MALAT1* a^36^re of significance when considering the increased rate of response of HDF. In addition to their protein content, exosomal *MALAT1* appears to enhance the effects of other cargo cytokines, miRNA, and lipids in wound healing.

## Innovation

Stem cell-derived exosomes allowed us to capture the power of stem cells for wound healing without applying cells directly to the wound. We found that ADSCs produced CM containing *MALAT1*, a lncRNA normally found in the nucleus. CM stimulated the healing of ischemic wounds *in vivo*, and cell migration in scratch assays and ECIS assays. *MALAT1* was packaged in exosomes in CM, and its uptake by wounded cells was demonstrated. The novelty of our finding is that ADSC secrete an exosomal cargo capable of stimulating HDF migration which is largely dependent on *MALAT1*, a lncRNA that normally functions in the nucleus for splicing of pre-RNA.

## Key Findings

Our study demonstrates that CM from hADSC contains exosomes with therapeutic properties:

- CM applied to HDF scratch assays stimulated HDF migration. CM applied to ischemic wounds also improved wound closure.
- CM contains *MALAT1*, a lncRNA normally found in the nucleus of cells that mediates alternative splicing of pre-RNA.
- *MALAT1* depletion of hADSC resulted in CM that failed to stimulate HDF migration.
- *MALAT1* was sequestered into exosomes in CM and mimicked CM in their ability to stimulate HDF migration.
- Hence, exosomes contain functional lncRNA that contribute greatly to their therapeutic applications.

## About the Authors

**Denise R. Cooper, PhD,** is a professor of Molecular Medicine, and former VA Senior Research Career Scientist, working in the areas of stem cell secretion, wound healing, and adipogenesis, with an emphasis on RNA splicing and function. **Chunyan Wang, PhD,** is a research scientist with interests in nanoparticles for targeting gene or drug delivery, hydrogel for tissue engineering, and nanoparticles for diagnostics. **Rehka Patel, BS,** is a research assistant with interests in drug development. **Andrea Trujillo, PhD,** is a research scientist with interests in the physiology of wound healing and the mesenteric lymphatic collecting ducts. **Niketa Patel, PhD,** is a VA research scientist and Associate Professor of Molecular Medicine working in the genetic signatures of adipose depots and delivery of exosomes for improving neurodegenerative disease. **Jamie Prather, MD,** is a general surgeon in La Crosse, Wisconsin. **Lisa J. Gould, MD, PhD,** is a plastic surgeon and combines her clinical practice in wound healing with clinical and basic science research on chronic wounds. She has served on the executive board of the Wound Healing Society for more than 10 years. **Mack H. Wu, MD,** is a surgeon and basic scientist. He is a VA Research Career scientist working in the signaling mechanisms of microvascular vasomotor and permeability responses to vascular endothelial growth factors and microvascular hyperpermeability during injury.

## Abbreviations and Acronyms

ADSC: adipose-derived stem cell
ASO: antisense oligonucleotides
bFGF: FGF2 or basic fibroblast growth factor
CM: conditioned media
ECIS: electric cell-substrate impedance sensing
EEC: endometrioid endometrial carcinoma
hADSC: human adipose-derived stem cells
HDFs: human dermal fibroblasts
iPSC: induced pluripotent stem cells
*MALAT1*: metastasis-associated lung adenocarcinoma transcript 1
MSC: mesenchymal stem cell(s)
MSCBM: mesenchymal stem cell basal media.
TBI: traumatic brain injury
TER: transcellular electrical resistance
